# Phylogeography of introgression: Spatial and temporal analyses identify two introgression events between brown and American black bears

**DOI:** 10.1038/s41437-025-00762-0

**Published:** 2025-04-19

**Authors:** Emily E. Puckett

**Affiliations:** Department of Biological Sciences, University of Memphis, Memphis, TN 38152 USA

**Keywords:** Evolution, Genetics

## Abstract

Brown bears (*Ursus arctos*) colonized North America from Eurasia in two distinct and temporally separated waves. Once in North America they encountered endemic American black bears (*U. americanus*) during range expansions from eastern Beringia southwards into the interior of the continent. The establishment of sympatry between these species provided the opportunity for hybridization and introgression, which was previously identified at the species level using *D-*statistics. Both species have broad spatial ranges that should limit the extent of introgression, such that it is found primarily between sympatric populations. Here, we used range-wide sampling and whole genome sequencing of both bear species to test for spatial variability in introgression. We identified two pulses of introgression between brown and American black bears, and demonstrate the introgressed segments occur across spatially structured lineages in both species. The first pulse occurred 270–120 kya, near the initiation of intraspecific divergence, approximately 99–93 kya, within each species. This pulse occurred as sympatry was established in western North America. The second pulse occurred between western American black bears and North American brown bears and lasted to 9 kya. Introgression was bidirectional and sympatric lineages had more introgressed tracts and a larger proportion of the genome introgressed from the other species. This study advances our phylogeographic understanding of both iconic bear species through investigating the timing of divergence and gene flow as bears expanded and contracted their ranges across North America.

## Introduction

**Fig. 1:**
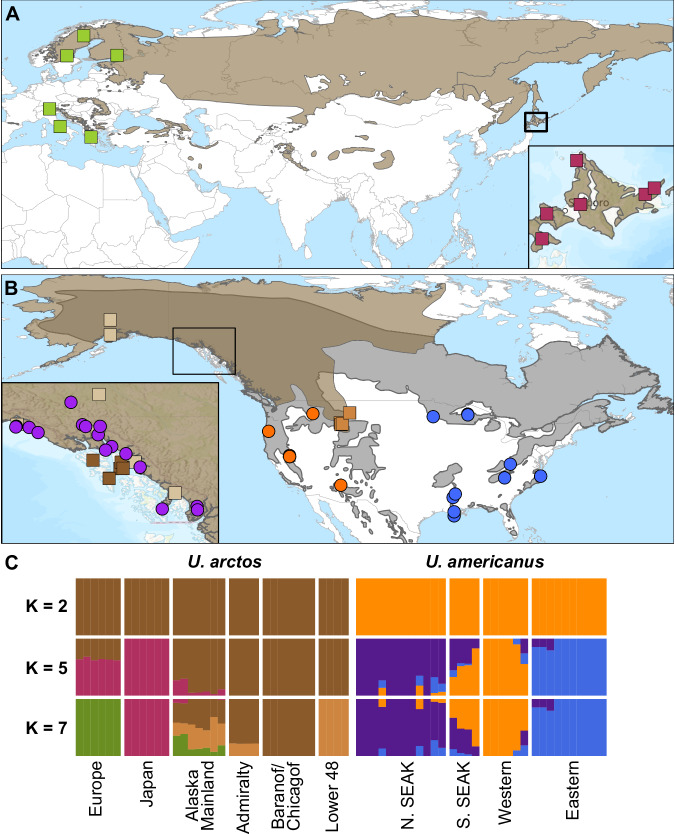
Sample distribution and two species ancestry clustering between brown (*Ursus arctos*; squares) and American black bears (*U. americanus*; circles). **A, B** Global range maps of brown (light brown) and American black (dark grey) bears with geographic locations of samples analyzed in this study shown as points colored based on broad ancestry from seven clusters across both species. Inset in panel **A** shows detailed view of Hokkaido, Japan, while the inset in panel **B** shows southeast Alaska, USA. **C** Clustering of all samples of both species showing distinct species clustering at two clusters (cross-validation shown in Fig. S2), the best supported model at five clusters, and seven clusters which were used as the units for most analyses throughout the paper. Single species clustering analyses shown in Figs. S3 and S4.

The bear family, Ursidae, originated in Eurasia with multiple extinct and extant species colonizing North America over its ~20Mya evolutionary history (McLellan and Reiner 1992). Within the subfamily Ursinae, there have been four colonizations of North America. The earliest was *Ursus abstrusus* (extinct) which either was conspecific to or shared a most recent common ancestor with *U. minimus* (extinct) (Kurten and Anderson 1980) the species which predominated across Eurasia. Called the primitive black bear, this species arrived in North America by 3.5Mya. The extant American black bear (*U. americanus*) evolved from *U. abstrusus* between 2–1Mya (Kurten and Anderson 1980). Contemporary genomic diversity of American black bears identified two nuclear lineages across the range (Puckett et al. 2015). The western lineage is found to the west of the Rocky Mountains, and extends south across the Mogollon Rim and into the Sierra Madre Occidental (Fig. 1B). The eastern lineage broadly expands from central Alaska, across the temperate forests of Canada, into the forested regions along the Atlantic seaboard, central interior highlands plateau, and southwards within the Sierra Madre Oriental (Pedersen et al. 2021; Puckett et al. 2015). The eastern lineage has a signature of isolation-by-distance as Alaskan subpopulations have a strong eastern signature (Bradburd et al. 2018) but have also been characterized as admixed with the western lineage, particularly in the southern portion of southeast Alaska (SEAK) (Puckett et al. 2015).

The second and third colonizations of North America were from the brown bear, *U. arctos*. Brown and polar (*U. maritimus*) bears are sister to the cave bear clade, and all of these species share a common ancestor with *U. etruscus* (extinct) (McLellan and Reiner 1992). Brown bears evolved in either central or northeastern Asia before expanding their range to both the west and east (Anijalg et al. 2018; Segawa et al. 2021). Fossil evidence from both the west/Eurasian and east/North American sides of Beringia indicate that brown bears colonized North America between 191–130 kya and again from 29–12 kya (Salis et al. 2022). This fossil data was paired with mitochondrial haplotypes from aDNA and shows that clade 4 mitogenomes comprised the first wave, while clades 3a and 3b comprised the second wave (Matheus et al. 2004; Waits et al. 1998). In brown bears, there appear to be four deeply diverged evolutionary clusters with additional hierarchical substructure across the range (de Jong et al. 2023; Tumendemberel et al. 2023). A cluster ranging from the Middle East across Europe towards the western side of the Ural Mountains comprises the first lineage (represented in this work as samples from peninsular southern Europe and Fennoscandia; Fig. 1A). The second lineage includes all North American populations except for the Kodiak Islands (hereafter North American; Fig. 1B). A third lineage has a longitudinal cline in European-North American ancestry when two clusters are distinguished and includes populations from east of the Ural Mountains across eastern Asia and the Kodiak Island (represented in this work with samples from Hokkaido, Japan; Fig. 1A). The fourth lineage includes samples from the Himalayan Mountains and Gobi Desert in central Asia. The fourth North American colonization was from the polar bear which has been in North America since at least 100 kya (Wang et al. 2022).

Among bears, post speciation gene flow has been identified for each of the six *Ursus* species and between clades within the phylogeny (Kumar et al. 2017). These analyses suggested both ancient gene flow mediated by extinct species, as well as more recent introgression events for species living in sympatry. Unidirectional introgression from polar into brown bears has been linked to a Pleistocene interglacial period 110–75 kya when species ranges became sympatric (Wang et al. 2022). Further, evidence shows that although polar bear ancestry varies between 3 and 9% within contemporary brown bear genomes, that introgression occurred into all populations (Cahill et al. 2018; Cahill et al. 2015). Brown bears on the Admiralty, Baranof, and Chichagof (ABC) Islands have long been recognized for their larger body size compared to other populations, and mitochondrial haplotype sequencing showed a close relationship to polar bears, sparking interest in this population. Subsequent genome scale analyses revealed that this was previously an isolated polar bear population that received continuous, male-mediated gene flow from brown bears since the last glacial maximum (LGM; 18–22 kya), and thus maintains polar and brown bear ancestry (Cahill et al. 2015).

Of the three North American bear species, viable hybrids from contemporary polar-brown matings are known from the wild and managed crosses (Miller et al. 2024; Preuß et al. 2009). All reported managed crosses used a male polar and female brown bear. Polar-brown hybrids are fertile as evidenced by births of captive F_2_ animals (Mann et al. 1957), and known captive and wild F_1_-polar backcrosses (Pongracz et al. 2017). Less is understood about viability and fertility of brown-American black hybrids due to fewer crossing attempts and available reports. Although F_1_ cubs are born, survival to sexual maturity varies. Contemporary fertility of brown-American black hybrids is unknown.

This study identifies spatial variability of brown and American black bear introgression. A previous test that identified introgression between these species used a single American black bear sampled in central Alaska, and two brown bears (European and ABC Islands) (Kumar et al. 2017). Given that brown and American black bears evolved largely in allopatry, the opportunity for these species to hybridize has been spatially and temporally limited. Spatially, these species have been and continue to be sympatric in western North America. While now extirpated, brown bears ranged as far east as the Great Lakes and into modern Labrador, Canada (Spiess and Cox 1976) with zooarcheological and aDNA dating their presence from at least 10–4 kya (Harington et al. 2014; Mather 2020). Temporally, hybridization would not have occurred until brown bears expanded their range into North America. The current data utilizing an American black bear from Alaska may or may not be representative of lineage specific patterns of introgression. We expect variability in introgression across species ranges based on the evolutionary histories of the taxa being compared. Here, we use range-wide sampling and whole genome sequencing of brown and American black bears to infer introgression patterns. We further estimated when in time introgression occurred and compared that to lineage divergence within each species to provide novel insight on the phylogeography of introgression.

## Methods

### Sample mapping to the American black bear reference genome

We utilized 32 *U. americanus*, 34 *U. arctos*, one *U. maritimus*, and one *Tremarctos ornatus* (Andean bear) whole genomes (Table S1) either by generating new resources or by downloading raw reads from the NCBI SRA. Genomes produced for this paper were constructed using 350 bp insert libraries with the NEB Next Ultra II DNA kit prior to sequencing on an Illumina NovaSeq with 150 bp paired-end reads. Library preparation and sequencing were conducted by Novogene (Chula Vista, CA). Reads were mapped to the American black bear reference genome (Srivastava et al. 2019) using BWA-MEM v0.7.17 (Li and Durbin 2010). We sorted, marked duplicates, then called variants (via HaplotypeCaller with the heterozygosity flag set to 5.0 × 10^−4^) using GATK v4.1.8.0 (McKenna et al. 2010). We removed the X chromosome scaffold (HiC_scaffold_1) from all analyses. For the longest 36 scaffolds in the reference genome (scaffolds 2–37; approximately 89% of the reference genome length), we built a database using GenomicsDBImport within GATK that contained all three species. From this database, we used GenotypeGVCFs for joint genotyping.

We quality filtered across all sites using BCFTOOLS v1.9 (Li 2011). Sites were included when: Fisher strand (FS) was less than 40, strand odds ratio (SOR) was less than 3, mapping quality (MQ) was greater than 40, MQ rank sum was between −5 to 5, quality of depth (QD) was greater than 2, read position rank sum was greater than −4, and depth across all samples was less than 5000 (depth cutoffs were set by calculating three times the mean mapping depth). We then filtered on the sites again removing those with individual sample depth less than 4, before filtering for genotype quality (QG) less than 30. From this set we retained biallelic sites (-m2 -M2), and removed indels (-v snps).

To create input files for MSMC analyses, each species was phased separately with BEAGLE v5.1 (Browning et al. 2018) using default settings and no imputation. Following phasing, samples were individually separated for input into the generate_multihetsep python script available as an MSMC tool. This preserved singletons within individuals. To create the positive mask files needed for MSMC, individual bam files had sites called using the BCFtools mpileup and call pipeline, with output exported to the bamCaller.py script. The vcf outputs were discarded but the mask files were retained. This dataset was used for all analyses, unless otherwise noted.

### Sample mapping to the ancestral *Ursus* genome

Given that a key question of this research relates to introgression, and post-speciation gene flow is prevalent among ursine bears, we repeated read mapping and genotype calling to a synthetic genome. We reconstructed the ancestral *Ursus* genome with the aim to reduce reference bias. We started from a multiple sequence alignment file (.hal) generated from 241 mammalian species by the Zoonomia Consortium (Armstrong et al. 2020; Genereux et al. 2020), then added in reference genomes for the sun (NCBI accession GCA_028533245), Asiatic black (GCA_009660055), American black (GCF_020975775), and brown (GCF_023065955) bears to the alignment (Pollard 2024) using Progressive Cactus (Paten et al. 2011a; Paten et al. 2011b). Code is available at https://github.com/mdpllard/bear_cactus. From this alignment, we exported a FASTA file containing the inferred ancestral reference for *Ursus* using hal2fasta. The synthetic genome had 3217 scaffolds and 2.24 Gb of sequence. We identified scaffolds syntenic with the human X-chromosome using hal2maf (Hickey et al. 2013), then removed from our analyses. We set a cutoff of 5 Mb as the length of a scaffold needed for inclusion in this study, which retained 1.93 Gb of sequence across 121 scaffolds.

We mapped a subset of samples (one representative from each geographic population) to the ancestral genome using BWA-MEM. Joint genotyping was completed using the mpileup function within BCFtools where the minimum mapping (-q) and base (-Q) qualities were set to 20, and the downgrade for mapping quality (-C) was set to 50. Output was directly piped to the call function where we implemented the multiallelic (-m) model and removed indels (-V). We quality filtered across all sites using BCFtools and retained sites when mapping quality (MQ) was greater than 30 and depth across all samples was less than 3000. We then filtered on the sites to retain biallelic SNPs. Finally, we used VCFtools v0.1.16 (Danecek et al. 2011) to remove sites with greater than 10% missing data, then randomly subsampled a site every 20 kb. This dataset resulted in 95,254 SNPs and was used for estimates of *D*-statistics (see below).

### Population structure

We first inferred lineage clustering within the samples in the dataset using PCA. We used VCFtools to remove outgroup species, thin the data to remove sites with greater than 10% missing data, and set the minor allele frequency greater than 0.05. We then randomly subsampled a site every 20 kb to reduce linkage disequilibrium among samples from the same within species lineage. This resulted in 108,478 SNPs. We repeated this process twice by subsetting the data into brown and American black bears before thinning sites. With each dataset, we ran a PCA in PLINK v1.19 (Chang et al. 2015). We further ran ADMIXTURE (Alexander et al. 2009) on for 20 iterations of on each of 1–25 clusters (K). The cross-validation (CV) error was plotted to identify the clusters with the lowest values indicating high support in the data for that number of ancestry groups. Notably, at the highest clustering values, the model identified single or pairs of individuals as clusters and were not considered robust estimates of clustering.

### Tests for introgression

We tested for signals of introgression between species using the qpDstat function within ADMIXTOOLS (Patterson et al. 2012). The *D*-stat test is arranged: (((p1, p2), p3), p4). We set the p1 and p2 taxa as individuals from the same species, either brown or American black bears; then the p3 taxon came from the other respective species. The p1 and p2 individuals either came from within the same intraspecific lineage or different lineages to test for geographic variation in introgression. The p4/outgroup individual was either an Andean or polar bear (Table S1) sample. For each (p1, p2), p3 test of interest, we calculated *D*-stats four times using a 2 × 2 factorial design, where the p4 species served as one factor, and the reference genome (ancestral or American black bear) was the second factor. This design allowed comparison among bioinformatic decisions for *D*-statistics. Notably, setting the p4 to the polar bear sample often violated the known topology of bears because this species is not a true outgroup; however, this was done to make comparisons to other works which use that topology. A 5 Mb block size was used for jackknife estimates to obtain Z-scores.

### Effective population size change through time

We estimated the change in effective population size (*N*_*e*_) through time using MSMC2 (Schiffels and Durbin 2014). We made a mask file for each sample with the bamCaller.py script which identifies genomic positions with high quality calls that were retained in the analysis. We also generated mappability masks for each of the 36 autosomal scaffolds in our analysis using SNPable (Li 2009) with a 35 bp mapping length and 50% stringency. Mappability masks identify genomic positions in the reference in which reads map uniquely, and are added into MSMC2 as a positive mask.

MSMC2 input files were made with the generate_multihetsep.py script. We ran MSMC2 on two samples (four haplotypes) from each focal population. For brown bears, this included four populations (northern Europe; Hokkaido, Japan; Admiralty Island, USA; and Lower 48, USA), and two secondary populations (southern Europe; Baranof/Chichagof Islands, USA). For American black bears, this included three focal populations (Nevada (western lineage); Appalachian Mountains (eastern lineage); and Yakutat, Alaska, USA (northern SEAK)). Previous work across SEAK has shown that the southern population is more similar to the western lineage (Puckett et al. 2015) which may be due to population extirpation and replacement (da Silva Coelho et al. 2023) after the LGM. Thus, we included southern SEAK as a secondary population for analysis.

### Population divergence timing

To estimate lineage divergence times within species, we input the cross-coalescent results from MSMC2 into MSMC-IM (Wang et al. 2020). MSMC-IM estimates gene flow between two populations over time. Within the model, gene flow is 0 in two scenarios: prior to population divergence and at the completion of population divergence. Thus, the model also results in estimates of the timing of population divergence, and the rate of differentiation. Further, plateaus in the migration probability (M(t)), indicate either admixture/introgression pulses between the two lineages being interrogated, or introgression into one lineage from a deeply diverged ancestor (Wang et al. 2020). All MSMC2 and MSMC-IM outputs were converted to years and number of individuals using a mutation rate of 10^−8^ (Kumar and Subramanian 2002). We varied the generation time depending on the species. Generation time of American black bears has been estimated at 6.5 years (Onorato et al. 2004), and brown bears at 10 years (Skrbinšek et al. 2012); thus, we used these values to scale MSMC and MSMC-IM estimates. We further used MSMC-IM to estimate migration timing in a set of cross-species comparisons, in which we took the mean generation time of 8.25 years per generation.

### Directionality tests of introgression

The MSMC-IM analyses indicated gene flow between brown and American black bears, yet does not indicate if this was uni- or bi-directional. We tested for introgression directionality using *D*_*FOIL*_ (Pease and Hahn 2015). *D*_*FOIL*_ is a five-taxon test able to test for introgression in the ancestral branches of a population tree. The test is arranged: ((p1,p2),(p3,p4),outgroup), where the p1 and p2 populations must have diverged more recently than the p3 and p4 populations. Based on our MSMC-IM results, we set European brown bears as the p1, the other three brown bear populations as the p2 individual, then western and eastern American black bears as the p3 and p4, respectively. However, as we predict gene flow occurred prior to contemporary lineage divergence in either species, we also flipped the positions of the species in the test; thus, American black bears were the p1/p2 and brown bears the p3/p4. We utilized our data mapped to the ancestral reference genome, and the single Andean bear sample served as the outgroup. Using three individual animals per group, 243 unique tests were run. The analysis was run in 100 kb non-overlapping windows (*n* = 12,115), and chi-square *P*-values were considered significant when less than 0.001. We required each window to have 400 sites (--mintotal) for inclusion in the analysis based on a divergence rate between the species of 0.4% (Cahill et al. 2013).

Using phased haplotypes from each species that were mapped to the ancestral reference and a subset of the samples, we used the program IBDmix (Chen et al. 2020) to estimate the percentage of each species which had been introgressed and retained to the present. Based on simulation data, false positives are greater for segments shorter than 30 kb (Chen et al. 2020); therefore, we limited segments to a minimum length of 30 kb and a LOD score greater than 4. Positions were exported into a bed file for each scaffold and each sample, then intersected with BEDTOOLS (Quinlan and Hall 2010) to remove duplicated introgressed regions across samples.

The length of introgressed tracks is related to both the recombination rate and time since introgression. Specifically, the expected length of an introgressed track (*L*) is 1/(*r* * *t*), where *r* is the recombination rate and *t* is the time since divergence in generations (Huerta-Sanchez et al. 2014). The recombination rate is unknown for bears, thus we used the estimate from domestic dogs of 0.97 × 10^−8^ (Wong et al. 2010). We scaled divergence time by a generation time of 8.25 years, splitting the difference between brown and American black bears. As an example, given the 30 kb cutoff of IBDmix, this method is expected to identify tracks: 1/(30,000 bp * 0.97 × 10^−8 ^bp per generation) * 8.25 years per generation = 28,350 years. A track more recently intogressed would be longer and captured by this analysis, but older tracks would not.

## Results

### Population structure within species

To confirm previously identified within lineage population structure ahead of grouping samples for analyses, we ran PCA across both species, and within individual species. The multi-species analysis clustered species separately (Fig. S1A, B), where the first axis contained 41.9% of the variation. Within the multi-species analysis, PCs 2–4 identified main axes of within lineage differentiation which were also present in the single species PCAs. PCAs based on species level variation identified expected geographic clustering (Fig. S1C–E). Brown bears produced three continental clusters representing samples from Hokkaido, Japan; northern and southern Europe; and across North America (Fig. S1C). Despite the unique evolutionary history of ABC Islands bears, they clustered with other North American samples, and separated by island on PC axis 4 (Fig. S1D). Within American black bears, the first PC axis produced an east-west split, while the second axis was along a north-south gradient (Fig. S1E).

ADMIXTURE analyses were largely confirmatory of previous work identifying rangewide or local population structure in bears. For brown bears, our ADMIXTURE results supported two evolutionary clusters between the eastern (Europe and Asia) and western (North America) hemispheres (Figs. S2B, S3). This best supported clustering was less than the six clusters identified from a rangewide analysis of brown bears with three-fold greater sampling than in this work (de Jong et al. 2023). Despite the lowest cross-validation support for two lineages, we analyzed four clusters: Europe; Hokkaido, Japan; ABC Islands, USA; and Lower 48, USA.

For black bears, our ADMIXTURE results supported three evolutionary clusters (Figs. S2C, S4). The eastern and western lineages were distinguished on the first PC (Fig. S1E) and at *K* = 2 (Fig. S4). Northern and southern SEAK clustered at *K* = 3 and 4, respectively. We selected four populations for further study: western lineage, eastern lineage, northern SEAK, and southern SEAK.

### Tests for introgression between species

**Fig. 2:**
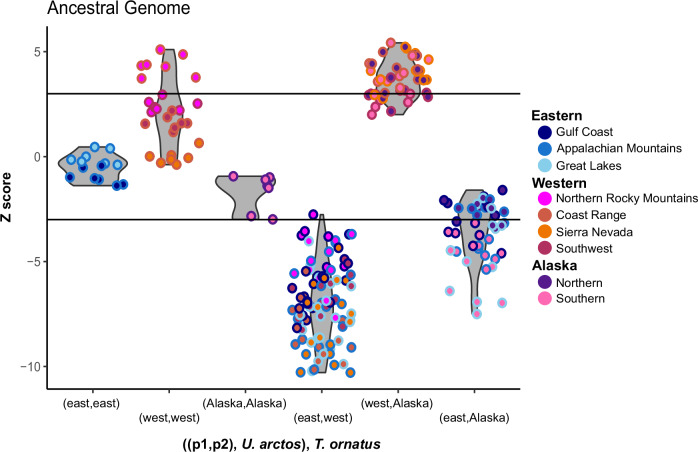
Tests of introgression between *Ursus arctos* (brown bears) and *U. americanus* (American black bears) using *Tremarctos ornatus* (Andean bear) as the outgroup and an ancestral *Ursus* reference genome for mapping. Colors represent the population of each animal used as the p1 (exterior/line) and p2 (interior/fill). Z-scores greater than 3 show significant introgression between the p1 American black bear and brown bear; where scores less than -3 indicate gene flow between the p2 animal and brown bear.

We tested for gene flow between brown and American black bears, and identified significant signals of introgression with western lineage American black bears (Figs. 2, S5). This pattern held regardless of the lineage of brown bear used as the introgressor. While all western lineage animals showed a signal of introgression when compared to the eastern lineage, when western black bears from different populations were compared, those from the Pacific coast (i.e., Oregon; Nevada) had stronger signals of introgression than the animal from the Northern Rocky Mountains; this is likely due to recent eastern lineage admixture into that population (Fig. S4). Further, animals from southern SEAK showed signatures of introgression when compared to eastern but not western lineage bears (Fig. 2), consistent with their admixed evolutionary history. To further explore introgression within this system, we tested for American black bear introgression into lineages of brown bears (Fig. S6). Significant introgression was observed between western lineage American black bears and the Lower 48 and ABC brown bears when European brown bears were the p2 population.

### D-statistic variability between reference genome and outgroup choices

For each *D*-statistic, we ran the same set of comparisons four times, varying the genome the samples were mapped to (ancestral *Ursus* or *U. americanus*) and the outgroup (*Tremarctos ornatus*/Andean bear or *U. maritimus*/polar bear). Given previous estimates of post-speciation gene flow among ursine bears, we consider the ancestral genome and use of the Andean bear outgroup as the best approach to test evolutionary relationships. When testing for introgression in American black bears (Fig. S5), there was high concordance between results from different mapping backgrounds. This pattern did not hold when testing introgression in brown bears (Fig. S6); specifically, more tests achieved significance with the species-specific reference genome than the synthetic ancestral reference. The choice of outgroup also impacted *D*-statistic estimation in this system. We recognize that polar bear is an inappropriate outgroup for tests between brown and American black bears due to phylogeny; however, we ran it to compare our results to other published *D*-statistics in the literature. Due to the incorrect phylogeny of polar bear as the *D*-statistic outgroup, there were more significant scores compared to using Andean bear regardless of reference genome (Figs. S5, S6).

### Effective population size change through time

**Fig. 3:**
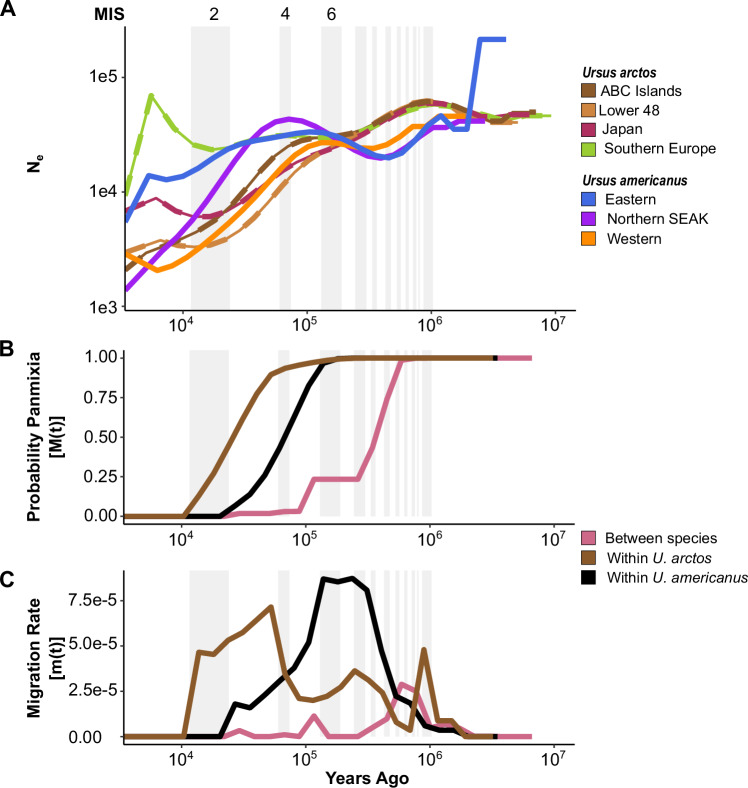
Intra- and interspecific patterns of demographic change among two bears. **A** Change in effective population size (*N*_*e*_) over time (estimated using MSMC2) in geographically structured populations of brown (*Ursus arctos*; dashed lines; green- southern Europe; maroon- Japan; dark brown- ABC Islands; light brown- Lower 48, USA) and American black (*U. americanus*; solid lines; blue- eastern; orange- western; purple- northern SE Alaska) bears. Lineage divergence patterns are shown as **B** the probability that two lineages are a single population decreases over time, and the rate of that divergence is influenced by the **C** bidirectional gene flow (estimated with MSMC-IM). Each line represents a comparison between two lineages, including: brown- Lower 48 and southern European brown bears; black- western and eastern American black bears; and rose- Lower 48 *U. arctos* and western *U. americanus*. Light grey background indicates glacial periods with labels for times described in the main text (left to right: Marine Isotope Stages 2, 4, and 6).

We estimated changes in historic *N*_*e*_ for lineages of brown and American black bears using MSMC2, then scaled the estimates to years using a mutation rate (µ) of 10^−8^ and a respective generation time of 10 or 6.5 years. Brown bear populations share a common pattern of *N*_*e*_ until population divergence initiates. Ancestral effective population size peaked at 60k around 1Mya then declined. Following divergence, both North American populations and the Japanese population experienced declining *N*_*e*_ during the late Pleistocene glaciations (Fig. 3A). The Lower 48 and Japanese populations appear to increase in *N*_*e*_ following the LGM; however, a similar increase was not observed on the ABC Islands. In contrast, the European population showed a unique pattern where *N*_*e*_ had minor fluctuations from 200–10 kya, then a substantial increase following the LGM (Fig. 3A).

Ancestral population size in American black bear populations was 40k approximately 1Mya, then declined to 20k by 400 kya (Fig. 3A). From 400 kya to the end of marine isotope stage (MIS) 5, *N*_*e*_ increased in the eastern lineage and SEAK populations. The western lineage began a steady decline at the end of MIS 5, whereas the eastern lineage began declining during the last interglacial (MIS 3) that continued through the LGM (MIS 2). The northern SEAK population declined rapidly during the last interglacial (Fig. 3A).

### Within species population history

We used MSMC-IM to investigate the patterns of lineage divergence and admixture within each species. We extracted the mean of the estimated split time to describe divergence patterns.

#### Brown bears

The earliest population divergence in brown bears was between the Lower 48 and Japanese populations approximately 92.8 kya (Figs. 3B, S7). Divergence between the European and either Lower 48 or Japanese populations proceeded shortly thereafter, at 90.0 kya and 76.5 kya, respectively. Finally, we observe the divergence of the ABC Islands population from European population around 59.3 kya (Fig. S7). The Lower 48 and ABC Islands diverged more recently than other populations, at 17.6 kya. Notably, divergence appears to begin at a similar time as divergence among other lineages but slows throughout MIS 2 and shows a marked increase in gene flow after the LGM (Fig. S7B). Similarly, divergence within the ABC Islands and within European bears was estimated at 6.8 and 5.6 kya, respectively due to gene flow being established following glacial ice retreat (Fig. S7A, D).

#### American black bears

The deepest divergence within American black bear lineages was similar to that of brown bears as the western and eastern lineages diverged around 99.2 kya; however, the rate of divergence was faster (Figs. 3B, S8). The eastern lineage and northern SEAK diverged around 36.8 kya (Fig. S8B, C). Divergence between the adjacent northern and southern SEAK populations was estimated at 8.5 kya. Notably, the migration rate curves between these populations (Fig. S8B) highlight the western and eastern lineage divergence (associated with southern and northern SEAK, respectively) and a second more recent migration pulse consistent with post-LGM gene flow within this geographic region.

### Species divergence and introgression

We compared MSMC-IM bidirectional migration rate curves between lineages of brown and American black bears. The mean split (i.e., speciation) time estimate was 736 kya (range from pairwise population estimates 686–861 kya; Figs. 3B, S9); however, the migration plot begins to show positive m(t) estimates from 3.7–2.5 Mya (Fig. 3C). A period of gene flow between all brown and both American black bear lineages was observed from 270–120 kya (Figs. 3B, S9). The corresponding migration rate goes to 0 at the transition from MIS 7 to 6, before increasing at the end of the glacial period and into the MIS 5 interglacial (Fig. 3C). The height of the plateau in the migration probability suggests the rate of introgression (Wang et al. 2020); thus, we estimate introgression between 17 and 25%, which appears as a longer duration event in the western than eastern lineage of American black bears. Gene flow signatures continued between brown bears and the western lineage of American black bears until about 9 kya. This pattern was not observed in eastern lineage American black bears. There was a 1% introgression between western lineage American black bears and the ABC population of brown bears (Fig. S9).

**Fig. 4:**
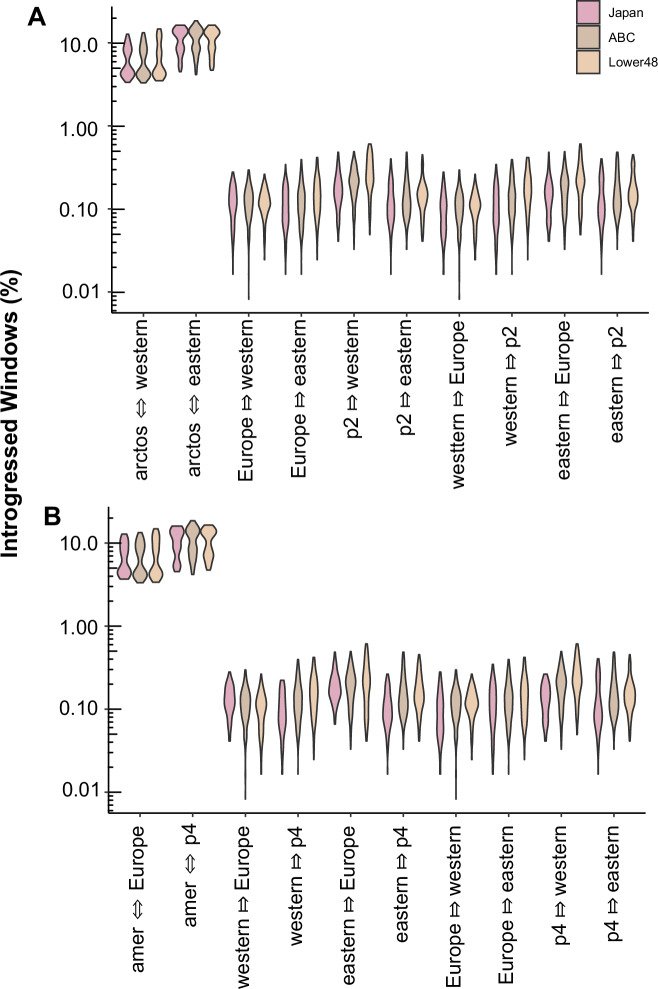
Introgression between brown (*Ursus arctos*; arctos) and American black (*U. americanus*; amer) bear populations estimated using *D*_*FOIL*_. Population order was varied due to estimates that lineage divergence initiated around the same time in each species. The five-taxon phylogeny was organized as: **A** ((*arctos*- Europe, *arctos*- other), (*americanus*- western, *americanus*- eastern)), *Tremarctos ornatus*; or **B** ((*americanus*- western, *americanus*- eastern), (*arctos*- Europe, *arctos*- other), *Tremarctos ornatus*. The *U. arctos* p2 or p4 population varied between Japan (maroon), ABC Islands (dark brown), and Lower 48 (light brown). Each independent run set a single animal in each position, and three animals from each population were selected and *D*_*FOIL*_ run for all four-way combinations. Violin plots were produced from the number of 100 kb windows which *D*_*FOIL*_ assigned to each introgression pattern divided by the total windows tested (*n* = 12,115).

Given the deeper time introgression signal within the two species MSMC-IM, we next tested if introgression was uni- or bi-directional using *D*_*FOIL*_ with 12,115 nonoverlapping 100 kb windows. Regardless of the orientation of the two species within the p1/p2 or p3/p4 positions, we observed the highest number of windows containing an introgressed signal between the ancestral population of one species approximately evenly divided into the two test populations of the second species (Fig. 4). We interpret this pattern as representative of gene flow occurring between the ancestral populations of brown and American black bears prior to contemporary lineage divergence.

To assess the proportion of the genome containing ancestry tracks from the other species, we utilized IBDmix. North American brown bear tracks comprised between 0.336 and 0.710% of the American black bear genome (Table 1). The total number of tracks and their cumulative length were greater when the candidate introgressor was a brown bear from the Lower 48 population as opposed to an ABC Island subpopulation. Similarly, American black bear tracks made up 0.091–0.740% of the genome in North American populations of brown bears. Western populations introgressed approximately eighth-fold more tracks into brown bears than eastern lineage populations. As described in the methods, the age of detected tracks is expected to be younger or equal to 28 kya and thus more representative of the more recent introgression event.

**Table 1:** Summary statistics of introgressed tracks between *Ursus arctos* and *U. americanus* estimated by IBDmix.

Population	*n*	Number of Tracks	Total Length (Mbp)	Percent
*Brown bear tracks within Black bear samples*				
Admiralty	3	133.3 ± 14.2	9.3 ± 0.9	0.484 ± 0.047%
Baranof/Chichagof	3	92.7 ± 11.4	6.5 ± 1.6	0.336 ± 0.082%
Lower 48	3	185.0 ± 10.4	13.7 ± 1.0	0.710 ± 0.050%
*Black bear tracks within Brown bear samples*				
Eastern	3	20.3 ± 7.8	1.7 ± 0.8	0.091 ± 0.044%
Southeast Alaska	4	68.3 ± 5.7	6.5 ± 0.4	0.336 ± 0.021%
Western	4	169.5 ± 28.4	14.3 ± 3.2	0.740 ± 0.164%

A subset of individual samples (*n*) from a population were tested as introgressors into samples from across the populations of the other species. Average and standard deviations are reported for the number of unique introgression tracks counted, the sum of all track lengths per individual introgressor, and the percent of introgression from that population into the other species. Samples were mapped to the *Ursus* ancestral reference genome to prevent bias.

## Discussion

Understanding the phylogeography of introgression encompasses examination of: shared species-level and unique lineage-specific patterns of introgression, quantifying introgression proportions, and inference about how spatial variation arose and was maintained into the present given a species evolutionary history. The study of human populations has identified shared and lineage specific gene flow with archaic hominins (Ahlquist et al. 2021), as well as demonstrated lineage-specific adaptive introgression (Huerta-Sanchez et al. 2014). The introgression phylogeography of maize and teosinte led to understanding the high genetic diversity of this important crop, and the background lineages upon which artificial selection has acted (Yang et al. 2023). These examples highlight how spatial variation in introgression can lead to novel inference, particularly for locally adapted traits. Understanding the phylogeography of introgression is not limited to model systems, and may be broadly useful for species occupying large and/or ecologically diverse ranges, or for closely related taxa where historic or current sympatry occur. Here, bears are used to investigate spatial variation in introgression and effort is made to understand when in evolutionary time gene flow occurred.

**Fig. 5:**
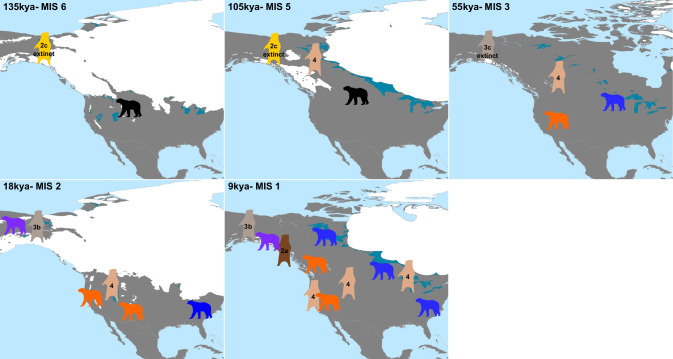
Schematic of American black (*Ursus americanus*; all-fours) and brown (*U. arctos*; standing) bears depicting occupation of unique lineages across North America over time. Each map shows water as glaciers (white), glacial freshwater lakes (peacock), and ocean (pale blue). The time stamps refer to the specific interpolated glacier layers (Dyke 2004) and associated Marine Isotope Stages (MIS). Bear pictogram color denotes the lineages of the respective species, where American black bears include: orange- western; blue- eastern; and purple- northern SEAK; and brown bears include: tan- North American lineage with clade 4 mitochondrial haplotypes; dark brown- North American lineage with 2a haplotypes; gold- haplotype 2c; and grey- haplotypes 3c (extinct) and 3b (extant although not discussed in this work).

Ursine bears have received considerable attention for post-speciation gene flow. However, single sample studies may over- or under-estimate the impact of introgression, particularly for species with large and structured spatial ranges. Due to the initial sequencing of an eastern lineage American black bear, and then a closely related Alaskan animal, introgression patterns from the western lineage have not been investigated. This study focuses on the phylogeography of introgression (Fig. 5), and particularly identifies two introgression pulses between brown and American black bears.

Our data suggest that an initial introgression pulse between brown and American black bears occurred at nascent stages of intraspecific divergence within each species (Figs. 3B, 4). The timing of this introgression event (270–120 kya) thus explains why signal is present across all brown and American black bear populations, particularly those that have never been sympatric. Specifically, insufficient time had passed between intraspecific lineage divergence and introgression, such that alleles fixed in populations today were still being sorted when introgression occurred. This timing is congruent with the fossil record for the first wave of brown bears coming into eastern Beringia between 191 and 130 kya (Salis et al. 2022) (Fig. 5).

Sympatry between brown and American black bears was likely established during MIS 5 (130–71 kya) when the former moved southwards (Salis et al. 2022) while the latter moved northwards. The western lineage of American black bears has older allele ages than the eastern lineage (Puckett et al. 2023), thereby suggesting that the contemporary eastern lineage is the result of population divergence and range expansion. The presence of brown bear alleles within eastern lineage bears suggests this range expansion occurred via a northern route (i.e., across modern Canada) instead of a southern route (i.e., across modern Mexico then along the Gulf of Mexico) where black bears would have encountered desert ecosystems. A northern expansion route also explains the isolation-by-distance signature between eastern lineage and Alaskan populations (Bradburd et al. 2018). Specifically, we propose that once the eastern lineage range expansion began, that bears traveled in eastern and western directions, which produces the unique phylogeographic history for this species. This hypothesis was supported by the divergence timing estimates of the eastern lineage (Fig. S8C) which indicated that the northern SEAK population initiated divergence rapidly after the eastern lineage split from the western (Fig. 5).

Our data show a second signal of introgression into the western lineage of American black bears (Figs. 2, 3B, S9). Here, the migration rate, m(t), increased between western black bears and all examined populations of brown bears between 90 and 9 kya. After the second wave of brown bears colonized eastern Beringia during MIS 4 (71–57 kya), they again expanded their range eastward and southward as the glaciers receded during MIS 3 (57–29 kya). However, it is unlikely that the second wave of brown bear colonization into North America strongly influenced the continued gene flow with the western lineage. Instead, we propose that it was the eastern lineage becoming allopatric, or having severely reduced sympatry with Great Lakes brown bear populations, that creates the contrasting introgression signals. Alternatively, the sharp decrease in *N*_*e*_ (Fig. 3A) in the Lower 48 brown bear population may have necessitated searching for heterospecific mates, under an assumption that the decrease in *N*_*e*_ was associated with a decline in census size. Support for this hypothesis comes from our estimates that the Lower 48 population has an elevated proportion of American black bear introgression as compared to other global populations (Table 1).

### Unidentified introgression

There are two contrasting interpretations for a plateau within an MSMC-IM plot, either that gene flow ceased for a time between the two populations of interest (with a corresponding migration rate of 0), or that an unsampled population introgressed into one of the lineages at that time (Wang et al. 2020). A distinctive plateau appeared within the divergence comparison between brown and American black bears (Fig. 3) which partially spans a period of no gene flow likely related to the glacial period in MIS 6 but also spans a period of positive gene flow (Fig. S9). Although our interpretation of ancestral gene flow is based on the concordance between the MSMC-IM and *D*_*FOIL*_ results, we must entertain the possibility of one of four alternative explanations. Both brown and American black bears likely had now extinct lineages present between MIS 8–6. First, in brown bears, now extirpated diversity was represented by the clade 2c mitogenomes which share a mean time to the most recent common ancestor (tMRCA) of about 360 kya (Salis et al. 2022). Second, in American black bears, clade B mitogenomes diverged from clade A about 1.07Mya (Puckett et al. 2015), and a corresponding nuclear component of this diversity has not been sampled contemporarily, although recombination may obscure signal. The contemporary geography of those mitogenomes suggests the potential for admixture of intraspecific lineages in Eurasia (brown bears) or North America (American black bears), respectively, prior to the first wave of brown bears expanding eastwards, and thus possible sources of unsampled diversity could have come from either species. Although there are unexplained migration rate spikes apparent in both species (Fig. S7B, C: Asian and North American brown bears; and Fig. S8A: Alaskan populations of American black bears), the deeper timing between 500 kya and 1Mya suggests these are unlikely to cause the long plateau observed in this study.

A third possibility is that another bear species introgressed into brown or American black bears. Given the geographic distribution of bears, Asian black bears (*U. thibetanus*) are a possible candidate, especially for introgression into brown bears. An earlier work identified introgression between an Alaskan American black bear sample and samples from both Korean and Japanese Asian black bears (Kishida et al. 2022). Although brown bear samples were tested for introgression, the use of polar bear as the outgroup likely obscured signal. Further, it is unclear how the homoploid hybrid nature of Asian black bears (Zou et al. 2022) affects *D*-statistics. An alternative third species is the polar bear, as Barlow et al., (2018) estimated an excess of shared alleles between that species and American black bears.

Fourth, there is the possibility that the difference in generation time between brown (10 yr) and American black (6.5 yr) bears creates a back-log of mutations within MSMC-IM which coalesce rapidly thereby creating the plateau (K. Wang, personal communication). The program was designed for intraspecific not interspecific comparisons, and thus the two species analysis violated an assumption of the model. This likely also contributes to the shallow interspecific divergence time between 582–730 kya (Figs. 3, S9). While this is within the range 500 kya – 1Mya reported based on hPSMC (Cahill et al. 2016), both estimates are too shallow based on *Ursus* biogeography (McLellan and Reiner 1992), and phylogenetic trees based on both mitochondrial (Krause et al. 2008; Puckett et al. 2015) or nuclear coding sequence (Kumar et al. 2017) alignments. We recognize that SMC approaches lose accuracy further back in time; and that MSMC2 estimates are based off of the density of heterozygous sites, thus the higher fixed substitutions between species may result in underestimates of the initiation and 50% relative divergence time for interspecific estimates. While our model violation may explain a portion of the pattern, the ancestral gene flow was also inferred via *D*_*FOIL*_ which was designed for interspecific inference.

### Intraspecific phylogeography

Our results extend insight into the phylogeography of both bear species. Decades of brown bear phylogeography were inferred based on mitogenome clades which show strong patterns of spatial structuring. de Jong et al. (2023) argue that those mitogenome patterns persist even with high amounts of gene flow from the nuclear genome due to the breakdown of ancestral population structure by recombination. The increase in information from the nuclear genome has resulted in the inference that the ABC Islands are a population which traces its ancestry to the first wave of brown bear expansion into North America. Specifically, we estimate that the ABC and Lower 48 populations diverged 19.3 kya (50% divergence), and had substantial gene flow from 10–1 kya (Fig. S7A, B). The ABC Islands are unlikely to represent the nuclear diversity introduced during the second wave of brown bears into North America. Instead, we argue that the admixture signature observed in samples from central (i.e., Denali and Kenai) and mainland southeast Alaska (Fig. S3B, C) represent the second wave, which was supported by detailed sampling across Alaska (de Jong et al. In Review).

In regards to American black bears, we estimated that the western and eastern lineages diverged around 99.2 kya. A previous estimate of mitogenome divergence between the eastern and western clade A haplotypes timed this divergence at 170 kya (range 250–120 kya) (Puckett et al. 2015). As the oldest dated American black bear fossils are credited to the Irvingtonian (1.9Mya – 250 kya) and were unearthed in modern Pennsylvania and Maryland, USA in eastern North America, our data suggests population replacement of bears in the east. We did not observe signals within the MSMC-IM analyses among American black bears to suggest that the expansion of the modern eastern lineage encountered established black bear populations and admixed following divergence from the western lineage. One interpretation is that no mixing occurred due to population extirpation; future investigations of stratigraphic data of *U. americanus* fossils should test if an ancestral population was extirpated in the east. This scenario would be similar to recent findings of extirpation and population replacement of bears in eastern Beringia (da Silva Coelho et al. 2023; Salis et al. 2022). In regards to population replacement in modern Alaska, we observed increased migration rates between both Alaskan populations and the western lineage in time segments between 900 kya – 1Mya (Fig. S8A). These migration signals appear to capture deeply coalesced alleles that admixed from now extirpated diversity. One possibility for the source of this deep coalescence, and the appearance only in comparisons with Alaskan samples, is that it represents the nuclear component of black bears containing clade B mitogenomes. The A and B mitogenome clades were estimated to have diverged 1.07Mya (Puckett et al. 2015), consistent with the migration peak. Deeper investigation of these alleles and how they may contribute to adaption in the northern-most extent of the range are warranted.

### Variation in D-statistics based on analysis choices

Our results highlight the multitude of ways in which experimental choices impact inference for introgression, including sample/lineage selection, outgroup selection, and reference genome choice. Inclusion of samples from across the focal species lineages contributed to how our results contrasted previous estimates of *D-*statistics and associated interpretations in the bear literature. In the first instance, we do observe introgression between American black and brown bears, in contrast to Cahill et al. (2013). Their work specifically compared ((ABC brown, GYE brown), eastern American black bear) and used the giant panda as the outgroup, a similar comparison to our work (Fig. S6). When we use the ancestral *Ursus* genome, no introgression was identified; however, when we use the American black bear genome, more similar to previous work which mapped to the polar bear reference, we observed variability in the significance of the tests. Variation between significant and not signals of introgression was due to the Lower 48 sample used, where the Yellowstone sample did not produce a negative Z score, concordant with Cahill et al. (2013). Thus, variation in samples and reference genome contribute variability to the results of *D*-statistics. Further, as we argue that the introgression occurred prior to brown bear population divergence within North America, the four-taxon *D-*statistic was not likely to capture any signal, as introgressed sites would be expected to have a BBBA pattern.

While this paper is the first to explicitly focus on introgression patterns between brown and American black bears, we are not the first to identify significant signal. Haplotype sharing was previously identified between an eastern lineage American black bear and North American brown bear subpopulations ranging from central Alaska southwards to Yellowstone, USA (e.g., Lower 48) (de Jong et al. 2023).

Most curious and concerning were the varying patterns of introgression observed due to changing the underlying reference genome. The use of the American black bear reference was in line with population genomic studies of introgression in other species in which a focal species is also used as the mapping reference (Cahill et al. 2013; Green et al. 2010; Rojas-Barrera et al. 2019). Concerns of mapping bias, where poor reference mapping of divergent species leads samples to look more similar to the reference in downstream analyses, may be an issue for some introgression analyses (Sarver et al. 2017). The generation of pseudogenomes, which introduce diverse sites into the reference genome through an iterative and/or reciprocal process (Huang et al. 2014), is one strategy to minimize reference bias. An alternative is the one presented here, which generates a reference genome from the ancestral sequence, thereby removing uniquely derived sites. As the ancestral sequence is shared equally among the focal species, reference bias should be reduced. The variation in inference on tests of introgression depending on taxa orientation and reference genome raises two salient questions. Are advanced bioinformatic mapping approaches needed for all systems? And if not, what characteristics of the study system suggest these extra measures are warranted for accurate inference?

Finally, we argue that by investigating introgression across a species geographic breadth, novel insight into evolutionary history is apparent. Introgression among these species is subtle and not readily detected through clustering methods (Fig. 1C). Future work will assess if introgressed tracks provide an adaptive advantage to either species, a particularly intriguing possibility in American black bears which show a longevity and fecundity trade-off between the intraspecific lineages (Beston 2011).

## Data archiving

Whole genome sequences have been deposited in the NCBI SRA under BioProject PRJNA867575. Code for expanding the Zoonomia Cactus alignment with additional bears is available at https://github.com/mdpllard/bear_cactus.

## Supplementary information

IntroPhyloGeog-Supplemental_v4.docx
