# Supplementary material for: Phylogeography of introgression: Spatial and temporal analyses identify two introgression events between brown and American black bears
Source: Heredity (Edinb). 2025 Apr 19;134(6):331–42. doi: 10.1038/s41437-025-00762-0 (PMC12137868; doi:10.1038/s41437-025-00762-0)
Supplement: Supplementary file 1 — IntroPhyloGeog-Supplemental_v4.docx [file 41437_2025_762_MOESM1_ESM.docx]

**SUPPLEMENTAL TABLES**

**Table S1-** List of samples used in this study. Metadata includes species, sample ID, geographic location, sex of the animal unless unknown (U), sample name in NCBI SRA, and original reference paper for group that generated the whole genome sequence.

|  |  |  |  |  |  |
| --- | --- | --- | --- | --- | --- |
| **Species** | **Sample ID** | **SRA Accession** | **Sex** | **Depth** | **Reference** |
| *T. ornatus* | Mischief | SAMN09225636 | F | 71 | Saremi et al 2022 |
| *U. maritimus* | PB3 | SRR15170761 | F | 16 | Miller et al 2012 |
| *U. arctos* | AKAdmiralty1 | [SAMN01057688](https://www.ncbi.nlm.nih.gov/biosample/SAMN01057688) | F | 37 | Miller et al 2012 |
| *U. arctos* | AKAdmiralty2 | SAMN02256321 | F | 13 | Miller et al 2012 |
| *U. arctos* | AKBaranof1 | SAMN01057689 | M | 36 | Miller et al 2012 |
| *U. arctos* | AKKenai | [SAMN01057690](https://www.ncbi.nlm.nih.gov/biosample/SAMN01057690) | F | 16 | Miller et al 2012 |
| *U. arctos* | ALP1 | SAMN07422272 | F | 18 | Benazzo et al 2017 |
| *U. arctos* | APN2 | SAMN07422262 | M | 18 | Benazzo et al 2017 |
| *U. arctos* | GRE2 | SAMN07422268 | M | 6 | Benazzo et al 2017 |
| *U. arctos* | AKAdmiralty3 | SAMN02045560 | F | 20 | Cahill et al 2013 |
| *U. arctos* | AKDenali1 | SAMN02045559 | F | 19 | Cahill et al 2013 |
| *U. arctos* | AKChichagof5 | SAMN03247209 | F | 9 | Cahill et al 2015 |
| *U. arctos* | JPHc1 | SAMD00282813 | F | 54 | Endo et al 2021 |
| *U. arctos* | JPHc2 | SAMD00282814 | M | 50 | Endo et al 2021 |
| *U. arctos* | JPHe1 | SAMD00282815 | F | 53 | Endo et al 2021 |
| *U. arctos* | JPHe2 | SAMD00282816 | M | 52 | Endo et al 2021 |
| *U. arctos* | JPHs1 | SAMD00282811 | F | 39 | Endo et al 2021 |
| *U. arctos* | JPHs2 | SAMD00282812 | M | 55 | Endo et al 2021 |
| *U. arctos* | AKBaranof2 | SAMN02256317 | U | 18 | Liu et al 2014 |
| *U. arctos* | AKChichagof1 | [SAMN02256316](https://www.ncbi.nlm.nih.gov/biosample/SAMN02256316) | U | 13 | Liu et al 2014 |
| *U. arctos* | AKChichagof2 | [SAMN02256318](https://www.ncbi.nlm.nih.gov/biosample/SAMN02256318) | U | 17 | Liu et al 2014 |
| *U. arctos* | AKChichagof3 | [SAMN02256319](https://www.ncbi.nlm.nih.gov/biosample/SAMN02256319) | U | 19 | Liu et al 2014 |
| *U. arctos* | AKChichagof4 | [SAMN02256320](https://www.ncbi.nlm.nih.gov/biosample/SAMN02256320) | U | 22 | Liu et al 2014 |
| *U. arctos* | OFS01 | SAMN02256313 | F | 15 | Liu et al 2014 |
| *U. arctos* | RF01 | SAMN02256315 | F | 15 | Liu et al 2014 |
| *U. arctos* | SJS01 | SAMN02256314 | F | 17 | Liu et al 2014 |
| *U. arctos* | MTgnp | SAMN02256322 | U | 17 | Liu et al 2014 |
| *U. arctos* | Adak | SRR19395449 | M | 45 | Armstrong et al 2022 |
| *U. arctos* | AK17500 | SAMN30214198 | F | 31 | Puckett et al 2023 |
| *U. arctos* | AK17512 | SAMN30214199 | M | 29 | Puckett et al 2023 |
| *U. arctos* | AK17578 | SAMN30214200 | F | 34 | Puckett et al 2023 |
| *U. arctos* | GYE906 | SAMN30214195 | F | 34 | Puckett et al 2023 |
| *U. arctos* | GYE922 | SAMN30214196 | U | 19 | Puckett et al 2023 |
| *U. arctos* | GYE953 | SAMN30214197 | U | 25 | Puckett et al 2023 |
| *U. arctos* | AK18626 | TBD | M | 20 | This study |
| *U. arctos* | YK46317 | TBD | U | 20 | This study |
| *U. americanus* | AK17023 | SAMN30214201 | M | 30 | Puckett et al 2023 |
| *U. americanus* | AK17047 | SAMN30214202 | M | 32 | Puckett et al 2023 |
| *U. americanus* | AZ12 | SAMN30214204 | U | 24 | Puckett et al 2023 |
| *U. americanus* | ID10 | SAMN30214205 | M | 32 | Puckett et al 2023 |
| *U. americanus* | NVb83 | SAMN30214212 | F | 36 | Puckett et al 2023 |
| *U. americanus* | NVg5 | SAMN30214214 | M | 37 | Puckett et al 2023 |
| *U. americanus* | NVb99 | SAMN30214213 | F | 33 | Puckett et al 2023 |
| *U. americanus* | MI334 | SAMN30214206 | M | 33 | Puckett et al 2023 |
| *U. americanus* | MI335 | SAMN30214207 | F | 34 | Puckett et al 2023 |
| *U. americanus* | MN6083 | SAMN30214208 | F | 15 | Puckett et al 2023 |
| *U. americanus* | MS3783 | SAMN30214209 | U | 29 | Puckett et al 2023 |
| *U. americanus* | NC056 | SAMN30214211 | F | 33 | Puckett et al 2023 |
| *U. americanus* | NC00417 | SAMN30214210 | F | 34 | Puckett et al 2023 |
| *U. americanus* | WV1701 | SAMN30214215 | M | 31 | Puckett et al 2023 |
| *U. americanus* | HA1 | SAMN30214216 | M | 15 | Puckett et al 2023 |
| *U. americanus* | HA2 | SAMN30214217 | F | 34 | Puckett et al 2023 |
| *U. americanus* | HA3 | SAMN30214218 | M | 39 | Puckett et al 2023 |
| *U. americanus* | HA4 | SAMN30214219 | F | 43 | Puckett et al 2023 |
| *U. americanus* | HA5 | SAMN30214220 | M | 41 | Puckett et al 2023 |
| *U. americanus* | HA6 | SAMN30214221 | M | 43 | Puckett et al 2023 |
| *U. americanus* | HA7 | SAMN30214222 | F | 43 | Puckett et al 2023 |
| *U. americanus* | HA8 | SAMN30214223 | F | 41 | Puckett et al 2023 |
| *U. americanus* | HA9 | SAMN30214224 | M | 39 | Puckett et al 2023 |
| *U. americanus* | AK17117 | SAMN30214203 | F | 35 | Puckett et al 2023 |
| *U. americanus* | AK18242 | TBD | F | 26 | This study |
| *U. americanus* | AK20340 | TBD | M | 22 | This study |
| *U. americanus* | AK20440 | TBD | F | 23 | This study |
| *U. americanus* | OR05 | TBD | M | 13 | This study |
| *U. americanus* | LA366 | TBD | M | 30 | This study |
| *U. americanus* | LAT593 | TBD | F | 26 | This study |
| *U. americanus* | LA371 | TBD | M | 30 | This study |
| *U. americanus* | YK48293 | TBD | M | 18 | This study |
|  |  |  |  |  |  |

**SUPPLEMENTAL FIGURES**

**
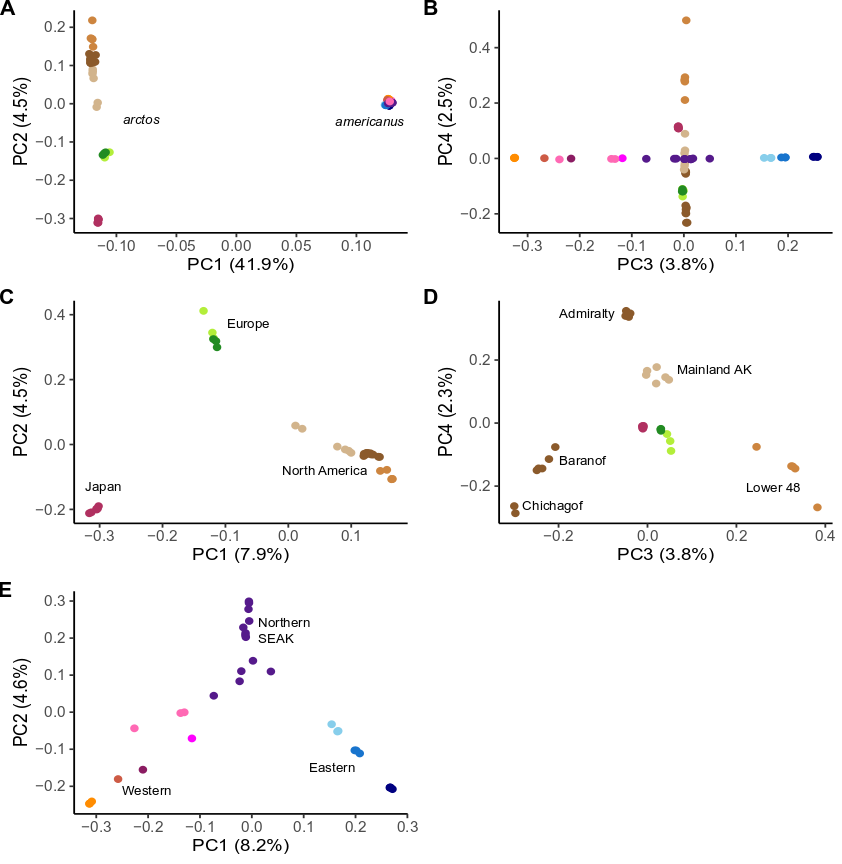
**

**Figure S1-** Principal components analysis of *Ursus arctos* (brown bears) and *U. americanus* (American black bears) samples. (A and B) The species separated along the first PC axis, with axes two and four identifying variation among brown bears, and axes three variation among American black bear. Within species variation for (C-D) brown and (E) American black were analyzed separately in PC space. Geographic descriptors used in the main text are highlighted near clusters of samples.


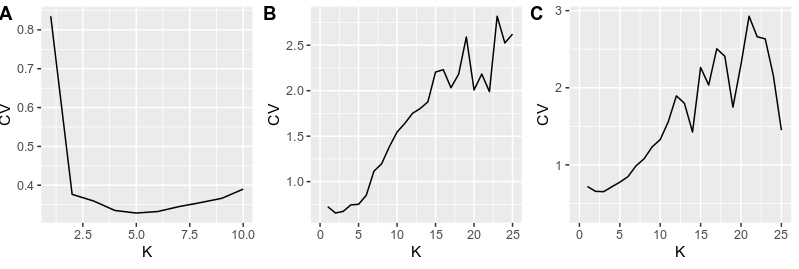


**Figure S2-** Cross validation error (CV) plots from ADMIXTURE for (A) *Ursus arctos* and *U. americanus*, (B) *U. arctos*, and (C) *U. americanus*. Clustering was run from 1 to 10 (two species) or 25 (single species) clusters (K) for 20 repetitions of the program to estimate cross validation error.


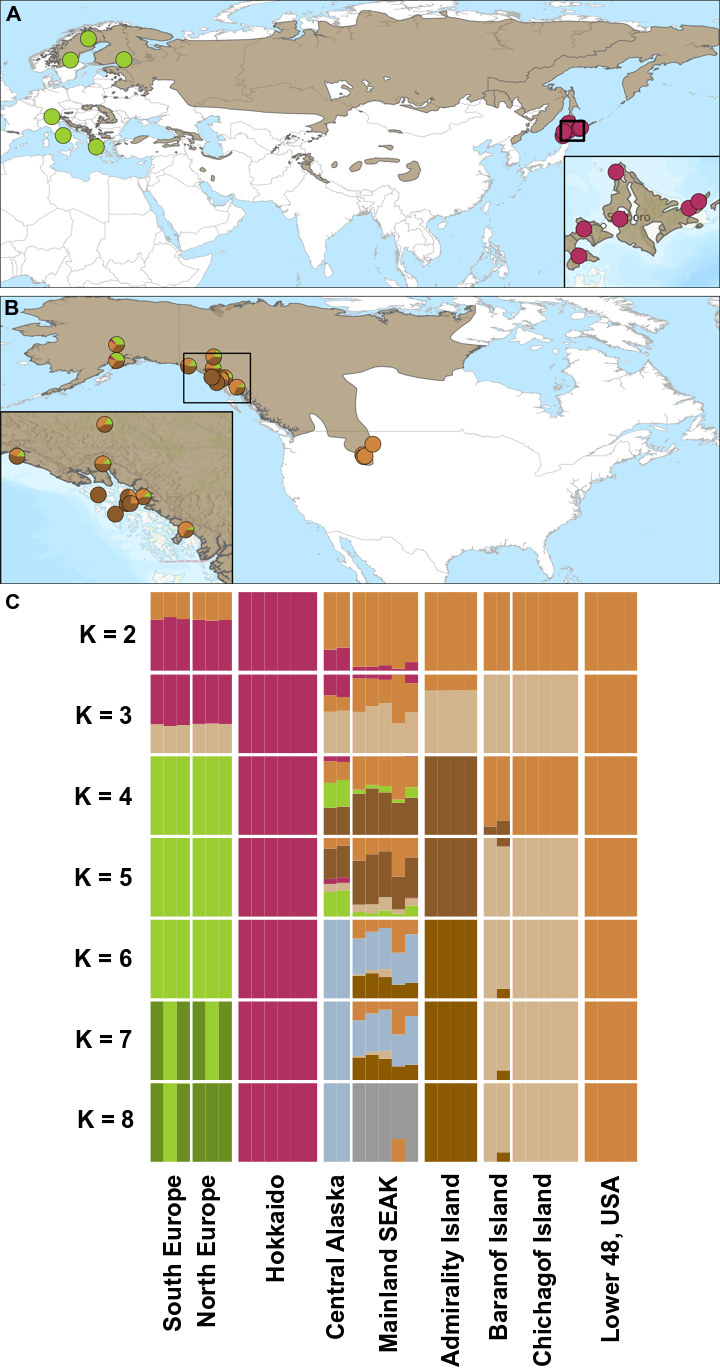


**Figure S3-** Population structure of *Ursus arctos* across 34 samples from around the globe. (A and B) Sampling locations of each bear shown as (C) ancestry proportions from four clusters (K = 4). Although two clusters were best supported by cross validation error (see Figure S2B), four clusters were discussed throughout the manuscript and thus displayed.


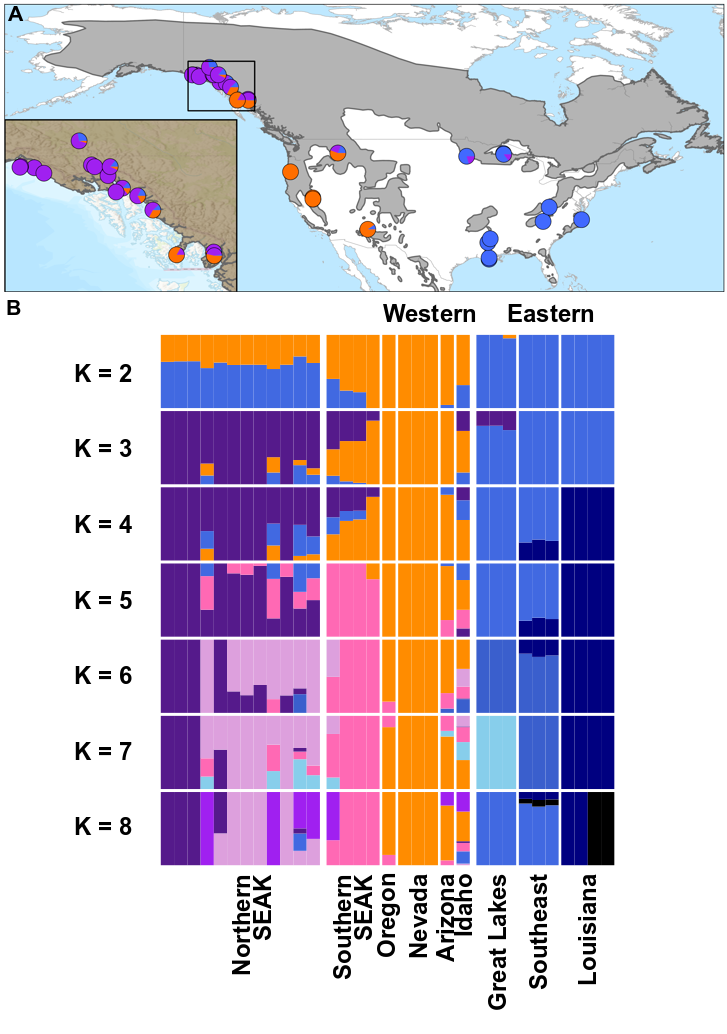


**Figure S4-** Population structure of *Ursus americanus* across 32 samples from around the globe. (A) Sampling locations of each bear shown as (B) ancestry proportions from three clusters (K = 3), which was the best supported model (see Figure S2C). The Southern SEAK population that appears at K = 5 was also discussed in the main text.


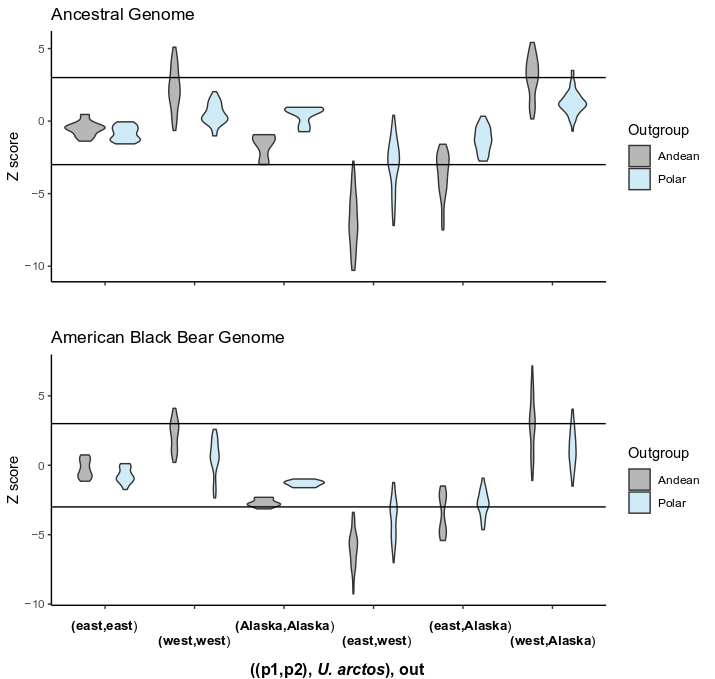


**Figure S5-** Variation in D-statistic estimates of *Ursus arctos* introgression into samples of *U. americanus* based on both the reference genome (top panel- *Ursus* ancestral; bottom panel- *U. americanus*) and choice of outgroup (grey: *Tremarctos ornatus*/Andean bear; ice blue- *U. maritimus*/polar bear). Z-scores greater than 3 indicate gene flow between the p1 and p3; whereas, Z-scores less than -3 indicate gene flow between p2 and p3. Tests are organized by the p1 and p2 *U. americanus* lineages for each sample (eastern, western, and Alaska).

**
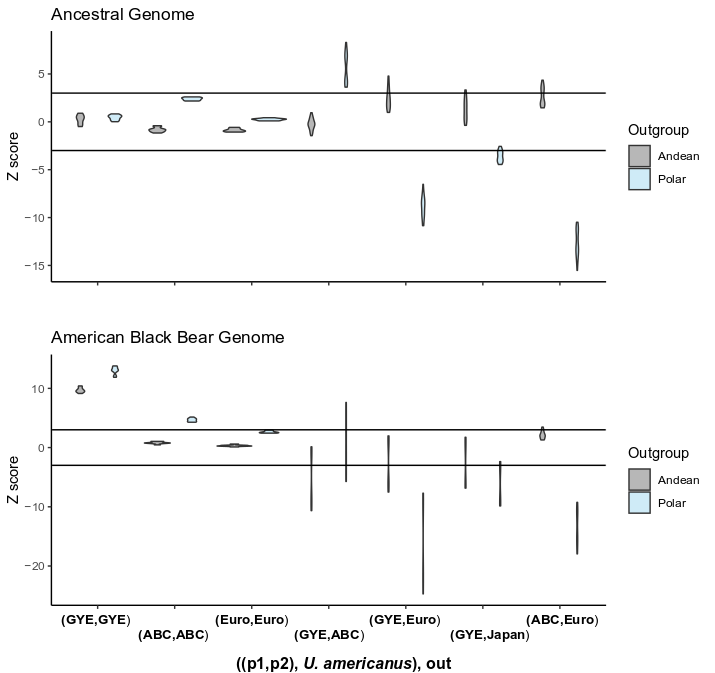
**

**Figure S6-** Variation in D-statistic estimates of *Ursus americanus* introgression into samples of *U. arctos* based on both the reference genome (top panel- *Ursus* ancestral; bottom panel- *U. americanus*) and choice of outgroup (grey: *Tremarctos ornatus*/Andean bear; ice blue- *U. maritimus*/polar bear). Z-scores greater than 3 indicate gene flow between the p1 and p3; whereas, Z-scores less than -3 indicate gene flow between p2 and p3. Tests are organized by the p1 and p2 *U. arctos* lineages for each sample (Greater Yellowstone Ecosystem, ABC Islands, Europe, and Japan).


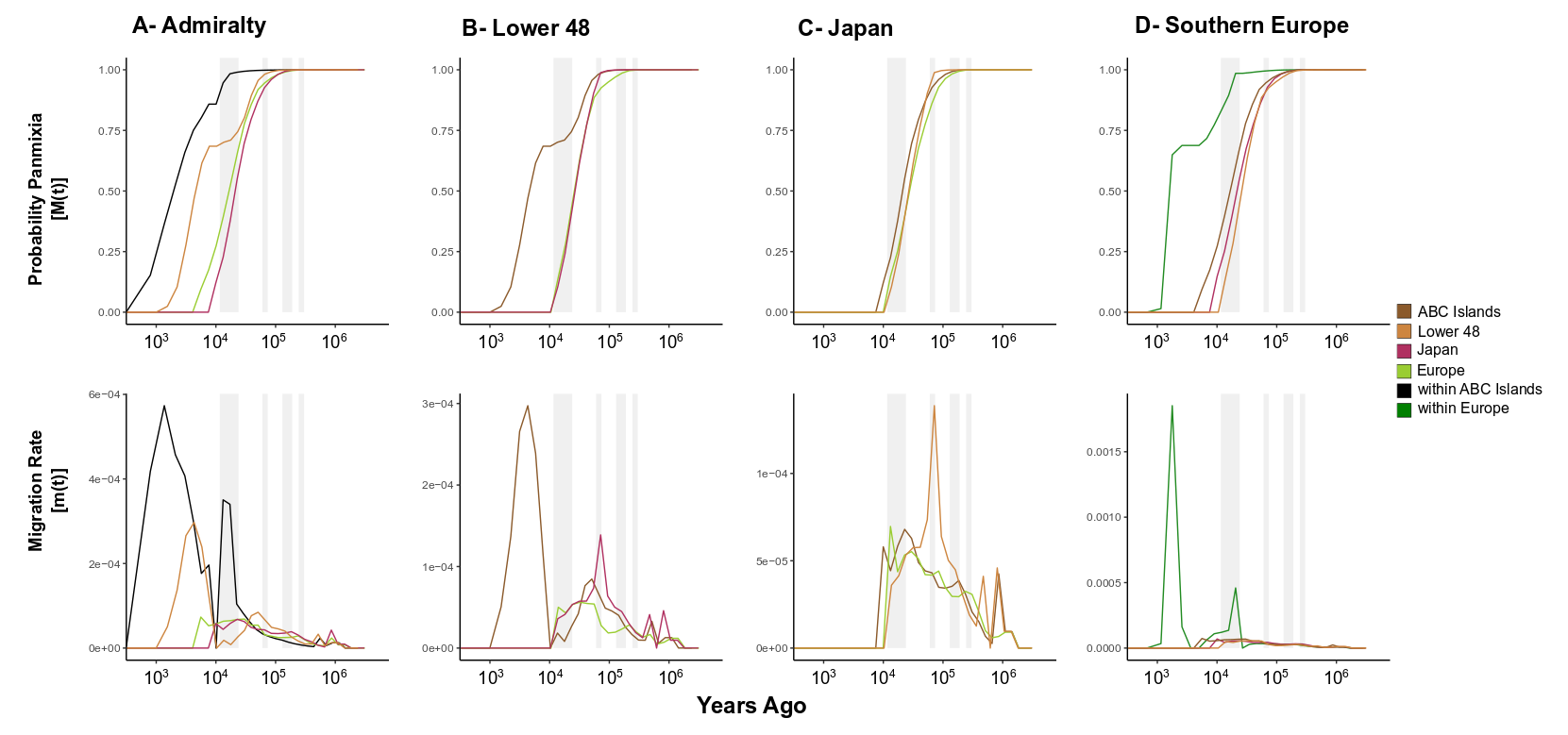


**Figure S7-** Population divergence among *Ursus arctos* populations through time. (Top Row) Change in the probability of divergence between the focal population (A- Admiralty Island, USA; B- Lower 48, USA; C- Hokkaido, Japan; D- southern Europe; n = 2 per population) and each other analyzed population (represented by the line color). All populations start as panmictic (100% probability of a single population), then go to fully diverged (0% probability of a single population). (Bottom Row) Estimate of bidirectional gene flow rate between two populations over time. Colors indicate second population in comparison against that listed (A-D), including: lime green- southern Europe; maroon- Hokkaido, Japan; dark brown- Admiralty Island, USA; light brown- Lower 48, USA. Additional comparisons in (A) between Admiralty and Baranof/Chichagof Islands shown in black; and in (D) between northern and southern European populations in medium green. Light grey background indicates glacial periods described in the main text (left to right: Marine Isotope Stages 2, 4, 6, and 8).


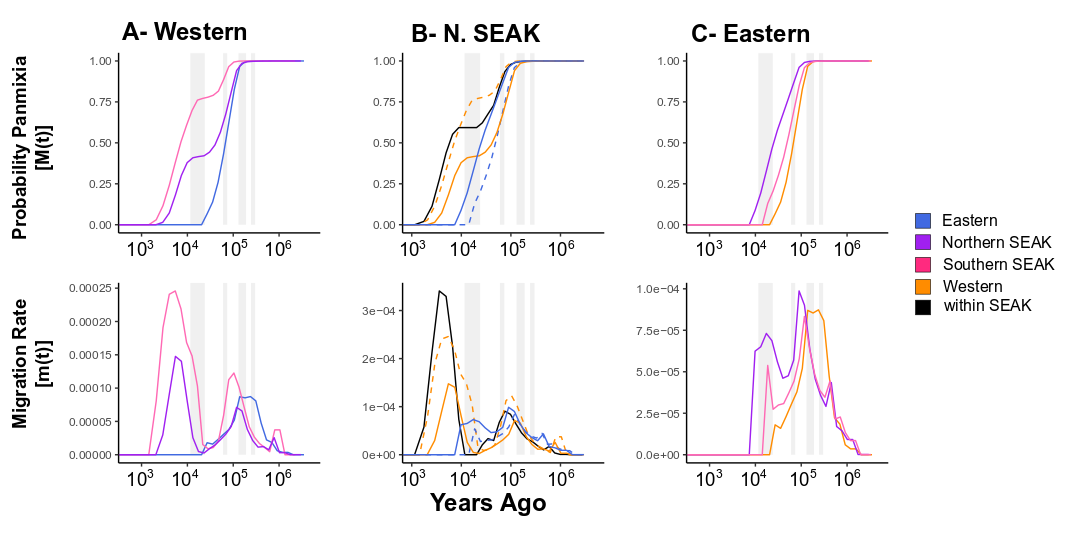


**Figure S8-** Population divergence among *Ursus americanus* populations through time. (Top Row) Change in the probability of divergence between the focal population (A- western lineage; B- northern SEAK admixed region; C- eastern lineage; n = 2 per population) and each other analyzed population (represented by the line color). All populations start as panmictic (100%), then go to fully diverged (0%) where gene flow no longer occurs. (Bottom Row) Estimates of bidirectional gene flow rate between two populations. Colors indicate second population in comparison against that listed (A-C), including: blue- eastern; orange- western; purple- northern SE Alaska; pink and/or dashed- southern SE Alaska. An additional comparison was made in (B) between the northern and southern SEAK populations (black line). Light grey background indicates glacial periods described in the main text (left to right: Marine Isotope Stages 2, 4, 6, and 8).


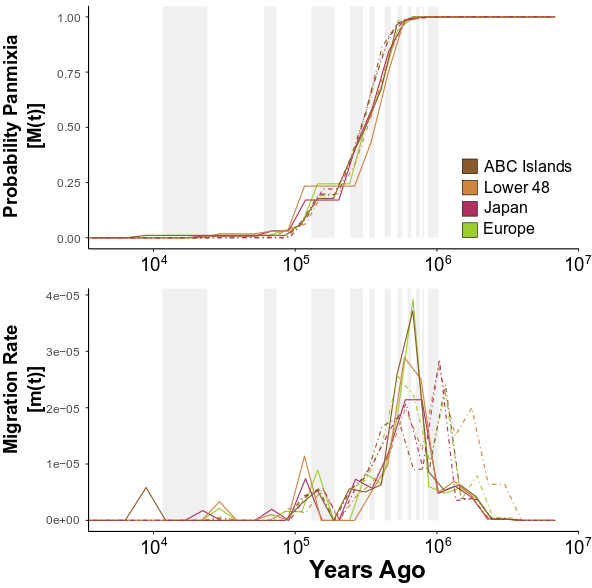


**Figure S9-** Divergence between *Ursus arctos* (colors: lime green- northern Europe; maroon- Hokkaido, Japan; dark brown- Admiralty Island, USA; light brown- Yellowstone, USA) and *U. americanus* (line style: solid- western; dashed- eastern) populations through time. (Top) Change in the probability of divergence over time. (Bottom) Estimate of the rate of bidirectional gene flow. Light grey background indicates glacial periods described in the main text (left to right: Marine Isotope Stages 2, 4, 6, and 8).
